# Gender aspects in parenthood conceptions of young Europeans. A critical reflection on the European Project B2-InF: A critical reflection on the European Project B2-inf

**DOI:** 10.12688/openreseurope.18210.1

**Published:** 2024-09-16

**Authors:** Joke Struyf, Kristien Hens, Virginie Rozée, Manon Vialle, María López-Toribio, José Miguel Carrasco, Vera Dimitrievska, Jana Meloska-Petrova, Anna De Bayas Sanchez, Michaela Fuller, Francisco Güell

**Affiliations:** 1 Philosophy, Universiteit Antwerpen Faculteit Letteren en Wijsbegeerte, Antwerp, Flanders, Belgium; 2 National Institute of Demographic Studies, Paris, Île-de-France, France; 3 APLICA Research and transfer, Madrid, Spain; 4 HealthGrouper, Skopje, North Macedonia; 5 Medistella, Prague, Czech Republic; 6 University of South Bohemia, České Budějovice, Czech Republic; 7 University of Navarra, Institute for Culture and Society, Navarra, Spain

**Keywords:** parenthood, gender roles, motherhood, fatherhood, LGBTQ+ parenthood, intersex fertility, trans fertility, family/career

## Abstract

The European research project “Be Better Informed about Fertility” (B2-InF) maps what young people understand about infertility and medically assisted reproduction (MAR) and the information they want or need. One part of this project were interviews with young people in eight European countries, which were analysed on legal, sociocultural and gender aspects. In this paper we present the gender analysis results focusing on gender differences in perceptions regarding the desire to become parents, social expectations versus parenthood as a choice, family/career issues and the division of care tasks, infertility, and the meaning of parenthood. The results demonstrate clear gender differences in parenthood responsibilities and appreciations. Traditional parenthood conceptions are strong: women are held responsible for reproductive issues, male infertility is a taboo and gender differences in care roles are distinct. Nevertheless, young Europeans often do not agree with these norms. Furthermore, while many participants are seeing increased social acceptance about LGBTQ+ parenthood, prejudices and legal obstacles continue to make LGBTQ+ parenthood difficult.

## Plain language summary

The European research project “Be Better Informed about Fertility” investigated what young people understand about infertility and medically assisted reproduction (MAR), and the information they want or need. We interviewed young people in eight European countries on issues like (in)fertility, parenthood and medically assisted reproduction. This article talks about their answers regarding parenthood, and the ideas on gender reflected in those answers. We write about whether gender influences the desires to become parents; whether young people feel they are pressured into becoming a parent or can choose to become a parent or not; the combination of family and career and the division of tasks within couples; and whether infertility and parenthood have different meaning for people of different genders. The results demonstrate clearly that men and women are often expected to have different parenthood responsibilities and parenthood has different meanings according to gender. Women are assumed to be responsible for reproductive issues, male infertility is a taboo and men and women have different care roles. Assumptions on gender differences also influence ideas regarding LGBTQ+ parenthood. While many participants see a growing social acceptance for LGBTQ+ parenthood, prejudices and legal obstacles continue to make LGBTQ+ parenthood difficult.

## Introduction

The European research project *Be Better Informed about Fertility* (B2-InF) maps what young people who have not already started a family understand about fertility and medically assisted reproduction (MAR). Part of this project were semi-standard interviews (n=97) with young people in eight European countries. Interviews covered themes like parenthood, infertility, MAR and knowledge about MAR. The interview data were analysed on legal, sociocultural and gender aspects. In this paper, we present the results of the gender analysis focusing on parenthood perceptions. The research questions are: What are young Europeans’ ideas on the gendered character of parenthood ( Bower-Brown, 2022)? Are women still perceived as the main responsible for reproductive issues, and if so, in which way? What do young Europeans think about the gender differences in care roles ( Pakaluk & Price, 2020; Yaffe, 2023)? With an increasing number of people using MAR ( Smeenk *et al.*, 2023) and deciding not to have children in Europe ( Beaujouan *et al.*, 2017; Colledge & Runacres, 2023), is infertility now less of a taboo subject? Where do young Europeans stand on the persistent stigmatization and discrimination against LGBTQ+ people and families?

## Methods

Project partners in collaboration with subcontracted companies conducted semi-structured interviews in eight countries: Albania, Belgium, Italy, Kosovo, North Macedonia, Slovenia, Spain and Switzerland. Between March and November 2021, ten to fifteen interviews were carried out with people between 18 and 30 years old, nationals or migrants living for more than 10 years in the country, who did not already have children or had used MAR. APLICA, a Spanish partner of the European research project consortium in charge of the fieldwork data collection, agreed with the companies subcontracted in Belgium, Italy and Switzerland on the sample definition and they recruited participants from their panels. APLICA conducted the interviews in Spain. Health Grouper, the partner in Eastern Europe, used snowball sampling to reach participants in Albania, Kosovo, North Macedonia and Slovenia.

Due to COVID-19 restrictions, many interviews were conducted via online platforms. ^1^ The sociodemographic characteristics of participants were collected in templates before the start of the interview. ^2^ All interviews were conducted in national languages, audio recorded, literally transcribed and translated into English. A code tree with themes and subthemes was decided upon by APLICA and HealthGrouper and validated by all consortium partners, and codes were assigned to all interviews using Atlas.ti. A first thematical analysis was performed by JMC and MLT on the interview data of Belgium, Italy, Spain and Switzerland, by VD for Albania, Kosovo and North Macedonia, and by a local researcher on the data from Slovenia. Afterwards, three teams ^3^ started analysing the data in depth from different perspectives: legal, sociocultural and gender. JS and KH conducted the gender analysis through reflexive thematic analysis ( Braun & Clarke, 2019). ^4^ We used NVivo and the original code tree to start from, but quickly established our own gender-related coding.

To decide upon which approach to use for the gender analysis, we used the checklist for integrating gender into health and biomedicine research design ( Schiebinger *et al*., 2021). We decided to investigate all issues regarding fertility, infertility, MAR and parenthood, connected to gender, sexuality and family form. We gave extra attention to LGBTQ+ parenthood for different reasons. Firstly, LGBTQ+ people are a large user group of MAR ( De Wert *et al*., 2014). Secondly, LGBTQ+ people are an important source of information about gender normativity, because ‘those who are not at home in categories tend to know more about them’ ( Ahmed, 2023, p. 141). Finally, non-LGBTQ+ participants’ conceptions about LGBTQ+ parenthood often shed light on implicit gendered assumptions (e.g. Bower-Brown, 2022).

During the analysis, we discovered that two participants in Albania and one from North Macedonia were already parents, so their data were excluded. Table 1 and Table 2 summarise the sociodemographic information concerning gender and sexuality of the participants included in the gender analysis, per country.

**Table 1.** Sociodemographic information of the participants concerning gender.

GENDER	Female	Male	Transgender MtoF	Transgender FtoM	Transsexual MtoF	Transsexual FtoM	Other	Total
Albania	6	3	-	-	-	-	-	9
Belgium	6	8	-	-	-	-	-	14
Italy	6	6	1	1	1	-	-	15
Kosovo	6	8	-	1	-	-	-	15
North Macedonia	5	3	1	-	-	-	-	9
Slovenia	5	5	-	-	-	-	-	10
Spain	7	7	-	1	-	-	-	15
Switzerland	4	5	-	1	-	-	-	10
**Total**	**45**	**45**	**2**	**4**	**1**	**-**	**-**	**97**

**Table 2.** Sociodemographic information of the participants concerning sexuality.

SEXUALITY	Heterosexual	Homosexual	Bisexual	Other	Total
Albania	7	2	-	-	9
Belgium	9	3	1	1 *	14
Italy	8	4	-	3	15
Kosovo	13	1	1	-	15
North Macedonia	7	2	-	-	9
Slovenia	10	-	-	-	10
Spain	7	4	4	-	15
Switzerland	5	4	1	-	10
**Total**	**66**	**20**	**7**	**4**	**97**

* asexual

To reach the goal of going ‘beyond the basic male/female dichotomy and also investigate, where possible, representations of and information about transgender and intersex populations’, the research team determined an ambitious aim for recruiting diverse profiles in the participant group. Nevertheless, intersex was not mentioned as a gender option in the sociodemographic information. It is estimated that 1,7% of the population is intersex ( Viloria, 2015), but we had no relevant participant information to use in the analysis.

The interview cohorts in the investigated countries were very different regarding the proportion of LGBTQ+ people. In Belgium, Albania and Slovenia no trans people could be recruited and in four countries only one trans person was part of the interview cohort. In Slovenia all participants are cis and hetero, while in Spain and Italy almost half of the participants identified as other than heterosexual.

In Table 3 the recruitment objectives considering the percentage of certain variables are put next to the actual percentage in the final analysis. While recognising that this research project succeeded in getting a diverse interview cohort, we notice that cis and heterosexual people are more present than planned.

**Table 3.** Diversity in the interview cohort in the recruitment objective vs. the final analysis.

VARIABLES	Recruitment objectives in %	Actual % in final analysis
**Gender** Female Male Other	**40** **40** 10	**46,4** **46,4** 7,2 *
**Sexuality** Heterosexual Homosexual Other	**50** 30 20	**68,0** 20,6 11,3 **

* Transgender MtoF (n=2); Transgender FtoM (n=4); Transsexual MtoF (n=1) ** Bisexual (n=7); Other (n=4)

## Results

Following the reflexive thematic analysis of the interview data, the following themes emerged: the desire to become parents; social expectations; family/career issues and the division of care tasks; infertility; and the significance of parenthood. Next, we present the results of the gender analysis regarding these themes.

### The burdened desire to become parents

Most participants state that they want to become parents in the future, but they feel it is still too early. The desire to become parents is often linked to concern: due to economic and ecological crises, participants doubt whether they can offer their offspring a good life. ^5^ A few participants talk about persistent patriarchal systems and one female participant worries especially about raising a daughter: ‘the issue of patriarchy… I would like to have a daughter, but what kind of world am I going to leave her in?’. 

In general, participants believe everybody, whatever their gender or sexuality, has a desire to become a parent. Some participants in Albania and Kosovo believe that desire to have children is stronger in women than in men. However, in our interviews female participants do not express a stronger desire to start a family than male participants. On the contrary, many participants know young women who do not look forward to becoming a mother, as well as people of all genders who emphatically do not want to become parents.

Many participants, LGBTQ+ and others, describe the difficulties LGBTQ+ people face in the process of becoming parents. First, they mention a biological obstacle: same-sex couples who want to have a biological bond with their child by definition need donor gametes and/or a surrogate. Gay male couples are considered to be in a less fortunate situation of needing a third person to carry their child while surrogacy is illegal or insufficiently regulated in all investigated countries. Participants assume going abroad to find a surrogate mother is possible but expensive. They are worried about the possible exploitation of surrogate mothers, i.e. the expectation that poor women would need to become surrogates because they need the money. Second, participants mention legal obstacles, for example in Italy a child can only have one legal mother, so if a lesbian couple separates, the other mother has no parental rights over their child ^6^. Moreover, in many countries LGBTQ+ couples are not allowed to adopt ^7^ ( ILGA Europe, 2023). Third, LGBTQ+ participants worry about the social attitudes towards LGBTQ+ parenthood. Some participants fear unfriendly reactions from doctors when they ask for information about MAR or parenthood. A female participant mentions a lack of information: ‘If we don't have a relationship with a cis man, or have a relationship with a cis woman, or with a trans man, at the moment of conception, how do we do it?’.

The greatest concern however is the fear their children would be bullied at school because of their parents’ gender or sexual identity. Heterosexual participants share this concern and name prevailing prejudices such as children of LGBTQ+ parents suffering from psychological stress due to the social reactions to their parents; gay parents raising their child to be gay; or children´s need of a father and a mother to ensure they develop properly. Some participants hold these views while others explicitly state that they do not adhere to these views.

 These biological, legal and social obstacles lead to many LGBTQ+ participants ruling parenthood out as an option while others have pushed it ‘to a faraway future’. Nevertheless, participants believe that people who have a hard time becoming parents, e.g. LGBTQ+ people or single parents, might be better parents: ’People that are fighting for it and really want it, they put in more effort afterwards for their child and his/hers upbringing. They are trying to do even better’. In Albania, Kosovo, North Macedonia and Slovenia, where participants were asked about involuntary or unplanned pregnancies, a similar idea was expressed: ‘Even when the pregnancy is not planned, the moment the couple has decided to take it to the end, it shows the desire they have to enjoy this moment’. Many participants believe it is important to think carefully before committing to start a family.

### Social expectations vs. parenthood as a choice

Participants agree that there is a societal expectation to have a family. From a certain age, people of all genders are stimulated to think about starting a family. The pressure of this socially dominant norm is felt especially in family surroundings, openly and in more subtle ways, e.g. parents of participants joking that ‘it’s almost time for [them] to become a grandfather or a grandmother’. One female participant mentions that even strangers feel entitled to ask about their plans to have children: ‘For example, I go to the store, and [someone might ask]: 'Where do you come from? Are you married? And you have no children? What do you mean, you have no children? When I was your age, I already had four’.

Participants in Italy, Slovenia, Spain and Switzerland express that this pressure mostly comes from older generations and family, and is more present in rural or religious communities: ‘[Childlessness is] an insult to the family. They say, why don’t you want to have descendants? We want our family to continue’. Some participants in these countries say there is no pressure to become parents soon or even at all within their own generation. One Spanish participant expresses that in her social circle, not having children is the norm: ‘the prospect of having a child is the antithesis’.

According to some participants, women are under more pressure to get children than men. This increased pressure is attributed to the fact that women’s reproductive life ends faster than men’s. The ’biological clock’ is often named: female participants do not want to ‘miss the opportunity’ and risk that ‘that ship has sailed’. Some participants believe male fertility diminishes as well but most are convinced that men can become fathers at a later age.

Across most countries, participants are often outspoken about the fact that men have more freedom in how they shape their lives. Some mention that men never get asked whether they want children, and a career or other accomplishments are viewed as worthy alternative life goals for men, while a childless woman is seen as lacking something, no matter her career or other accomplishments. A female participant from North Macedonia expresses that ’a girl should not become a woman but automatically become someone’s mother or wife’. Other participants call these gender differences ‘backward’ or ‘old fashioned’ ideas which are oppressive to women.

The expectation to have children is often posited as a social norm which an individual can conform to or rebel against. In all countries, participants believe that parenthood should be a choice. Nevertheless, participants in Kosovo, Northern Macedonia and Spain say that social pressure may prevent people from really making a choice. They blame religious precepts or the internalisation of social norms. One homosexual participant from North Macedonia says ‘the traditional lifestyle becomes so powerful that people don’t decide on things, but just do them routinely’, which he perceives as problematic.

 Nevertheless, participants also mention that young people carefully plan the ideal moment to start a family. Most important is to first reach economic and financial stability or certain career goals, which are personal goals for some, but social expectations according to others: ‘more is expected’ besides parenthood. A few participants mention the expectation to first find the right partner. Often participants emphasize the need for a certain degree of maturity before becoming parents, they want to be ‘fully developed’. They feel strongly about it, as this Kosovar participant expresses: ‘If I do not manage to meet all these criteria, I do not see it as reasonable to bring to life a person who cannot be offered all he needs’. However, some admit that finding the right time is difficult and the multiple societal and personal expectations ‘do not leave a place for a child’.

### Family/career issues and the division of care tasks

Participants in all countries worry about economic and financial stability. At the same time, mainly women worry about the combination of family and career. This combination was not part of the interview questions but many participants raised the issue spontaneously.

Participants mention gender differences in government and company policy, encouraging mothers to stay at home longer than fathers. They mention that women are blatantly asked about their wish to start a family in job interviews, less hired because they are assumed to become pregnant or fired once they are. Some describe the persistent belief that women cannot have a career and also have children, and the fact that the work environment is not adapted to new mothers. Although many participants wonder how to plan their family around or next to their career, especially women have to deal with this combination. Female participants worry about ‘paralysing their working life for a number of years’ in the middle of their professional carreer, or fear it will become practically impossible to continue their preferred job once they have children. The need for other caregivers with whom to share the care for their children is addressed by some participants. One mentions that caring for children has become an individualized task: ‘There is no longer that community of grandparents, or the neighbour who comes down to help you take care of the children’. Another participant wishes to raise or have children in a community or a commune. Furthermore, some female participants worry whether postponing parenthood increases fertility problems in women.

While many participants think people of all genders are capable of caring for children, they believe a gendered task division persists in heterosexual couples. One male participant from Belgium states that ‘the father is sometimes a little side-lined’ which he thinks is a ‘pity’. Others believe that ideas about the gendered task division are shifting and men are increasingly getting involved. Nevertheless, participants believe that mothers continue to spend more time with their children. In all countries, it is common for mothers to stay at home longer after the birth than fathers: they ’spend most of their time inside’ or are ’forced to stay at home’. When a child gets sick, it is usually the mother who takes sick leave and not the father. Some participants are bothered by the fact that as opposed to women, men are seldom asked about the balance between their work and being a father. Many participants do not agree with the unequal task division and think parents should share responsibilities.

### Infertility: a taboo for men and a responsibility for women

Infertility is perceived to be a sensitive topic which is often hidden and causes embarrassment, shame and feelings of inferiority, regardless of gender. Not knowing their fertility status leads to uncertainty and fear, and some participants wish to get their fertility tested, often prompted to do so by their peers or family.

Male infertility is considered a taboo in all countries. Participants indicate that in men, infertility hurts their virility, masculinity, ‘male ego’ or ‘male pride’. An infertile man is often perceived as ‘not a man’ and risks losing the respect or trust of other men. Several male participants blame this taboo on a toxic macho culture. Heterosexual male participants worry about their seed not being strong or healthy, because they are responsible for ’adding more people to the family’. They do not want to be seen as someone who ‘does not know how it is done’: ’you wish others to know that you are able to make a family of your own’. Should they be infertile, they would want to compensate that: ’I would need to show more of my masculinity in society’.

Men are not expected to talk about infertility because that would be seen as weak, which is why men suffer more from infertility, according to some participants. In some cases, when the interviewer asked about male infertility, the participants talked about female infertility instead. Women are assumed to talk about infertility problems with their friends and close family and to have a support network which helps them process the emotions and disappointment of infertility ( Taebi *et al*., 2021). Nevertheless, participants also often express the assumption that infertile women are suffering individually in silence. 

Many participants agree that women get blamed for the infertility of a couple, as ‘control and responsibility about reproduction normally corresponds to women’. Three female North Macedonian participants mention that it is common that women are sent to different doctors and have to undergo several examinations before the men get tested. Various male participants admit that they would assume their female partner was the source of their problems to conceive. Often men are more willing to divorce their spouse and look for a new partner than undergoing fertility tests themselves. Several participants in North Macedonia, Slovenia and Kosovo believe the risk of divorce for this reason is high.

Male and female infertility are expressed to be different, especially by participants from North Macedonia, Italy, Albania and Kosovo. In these countries, participants mention that ‘male infertility is seen as bad luck, while female [infertility] is almost a fault, a shame’ or ‘a curse from God’. Male infertility seems to be a stronger taboo than female infertility, but the links between ‘woman’ and ‘mother’ are also tight: woman’s ’whole function lies in her reproduction’ and a childless woman is ’not 100% a woman’ or ’a non-woman’. One participant wonders what women would do during the day when they are unemployed and have no children. Two participants mention that women are supposed to ‘enable a man to have offspring’ and therefore infertility is a bigger burden for women. Moreover, they believe that female infertility is harder to solve than male infertility, because a sperm donor is more easily found than a surrogate.

Two trans male participants demonstrate a lack of knowledge about trans fertility options: ‘Being a trans guy, the infertility part is not about me, whether I was fertile or infertile… It is something that is valued only in my partner’; ’I might not be as fertile as I thought I was’. This worry about fertility is reflected especially in trans participants and gay men living in Italy, North Macedonia and Kosovo.

### The significance of parenthood is gendered

Remarkably, in countries where parenthood is perceived less as an obligation or tradition – especially in Belgium, Italy and Spain ^8^ – participants use fewer words and go less into detail to describe what parenthood means for them. In countries where parenthood is seen as an obligation and an important role to fulfil, participants use more words to describe what it means to them, i.e. ensuring physical and psychological well-being, providing education, passing on values and creating a home. Parenthood is strongly connected to responsibility in Albania, Kosovo, North Macedonia, Slovenia and Switzerland. In Albania, Kosovo and Slovenia participants connect parenthood to maturing or becoming an adult, both as a requirement to becoming a parent as well as a consequence thereof. Continuing the family name or passing an inheritance are specifically named as roles of the father. In Kosovo and North Macedonia, reciprocity is mentioned as an important factor: parents take care of their children, and when participants are old, their children will take care of them. Therefore, some participants believe that voluntarily childless people will come to regret their decision later in life: ‘you’ll need [children] when you get older’.

For some participants, there is a clear difference between motherhood and fatherhood, especially in Albania, Kosovo and North Macedonia. Both mothers and fathers are important role models for their children, but it is the fathers’ job to ‘convey the right values’. Mothers are thought to be more ‘naturally’ caring, especially good with newborn children, and ‘more compassionate’. Men are assumed to be ‘rougher by nature […] and don’t express their emotions as much’. According to some, fathers allow more risky activities while ‘mothers are more protective’. Mothers are there ‘to take care of school, to tidy up […] while fathers are more for the cute things, including free time, sports activities, board games’. Mothers are supposed to take more responsibility for the upbringing and education of their children than men and spend more time with them. Men can choose to take responsibility for their offspring but are not judged badly if they don’t. For many participants, being a father means first and foremost to provide for the family via economic stability.

Fatherhood is less defined in Belgium, Italy, Spain and Switzerland. One participant thinks fatherhood is complicated and ‘generally thought of as more of a problem, it has more negative than positive connotations’. Assumptions about men’s (in)ability to care for children become apparent in expressions about gay male parents: they ‘wouldn’t really know what to do or what kind of responsibility they have’, while in lesbian couples, according to another participant, ‘[parenthood] is seen as much more natural, when in reality it isn’t’. Gender roles in parenting are also assumed in the expression that when a child has only one parent, ‘he [sic] tries to do both roles’.

Many participants think that in heterosexual couples, both the biological processes of pregnancy and birth and the unequal division of care tasks mean that mothers carry the heavier burden of parenthood. Some describe a mother’s task as a sacrifice. A North Macedonian participant mentions that mothers are expected to cut communications with others. Others express that fathers give up less on their hobbies while mothers leave their career, give up all pleasures and cannot spend as much time with their friends anymore. According to some, the physical, hormonal and mental changes during pregnancy make it difficult for a woman to have a child. It is ‘a little more hard-core’ and emotionally more intensive than a man’s experience. One participant mentions that he has several friends who don’t want to go through the pregnancy process.

The length of a pregnancy and breastfeeding period are set against the fact that men only need to be minimally involved in the conception. One participant describes that ‘men literally sow themselves’ and can have multiple children with several women without having to give in on their freedom. On the contrary, the biological implications for women mean their parenthood is much less non-committal. Some participants use biological involvement as an argument to explain differences in care obligations towards the child. While some emphasise that ‘it doesn’t mean the man shouldn’t be more involved’, for others, a father’s care and education responsibility only becomes equal to that of the mother ‘when the child is 4–5 years old’.

Many participants believe that a biological bond is established between mother and child during the pregnancy, some ‘synchrony’ which makes mothers ‘even more attached to the baby’. They call this a ‘maternal instinct’ or a ‘natural emotional connection between mother and child’. The maternal instinct is believed to be so strong that it is ‘enough to overpass the feeling that you don’t want the child’ in case of an unwanted pregnancy. Participants believe that a father ‘never has that relationship’: there is no ’fatherly feeling, or they hide it well’.

Whether they endorse the idea or not, many participants agree that there is still a strong ideal of a family consisting of a man and a woman and a few biological children. Single or LGBTQ+ parents are judged for not conforming to that ideal: ‘the community requires for two parents, a father and a mother’. Nevertheless, parenthood is considered more common in lesbian couples than in gay male couples: ’The two women have been taught that they are going to have children, so [parenthood] is seen as much more natural’.

Many participants believe that traditional gender divisions in parenthood are unfair and should change, with many believing that a change is already taking place. Some participants react strongly and call a gendered view on parenthood old-fashioned or retarded. Some think it is harmful for children’s development to be taught a gendered world view, which they connect with a general lack of openness towards LGBTQ+, infertility or other religions. Participants often believe that alternative family forms can be equally beneficial for children and that the acceptance of these alternatives is growing.

For some participants, parenthood is not related to gender at all. They stress that the essence of parenthood is being there and taking on the care tasks needed to raise children. ‘Being there’ is seen as essential which makes some participants critical of absent fathers: ‘If [the baby] is also part of you, then you have to be there. It’s not about helping, it is about being there’. Aunts and grandmothers have important roles to play, and according to some participants, a caring bond could be extended to relationships outside the family, through foster care, voluntary work or teaching. One participant says that ‘even if women don’t want to be mothers, that does not mean that they reject the importance of upbringing, transmitting values or pedagogy, or the whole process of accompanying a child’. One participant talks about the ‘house mothers’ in the 1980s New York ballroom scene, where people (typically trans women) took responsibility over homeless LGBTQ+ youth who were not accepted by their own families, considered them ’as their own children and offered them a roof over their head, the tasks that a parent should typically do’. Regardless of gender, participants are often positive about adoption, and many prefer adoption over MAR. Remarkably, compared to their ethical concerns with surrogacy, participants do not automatically link adoption with ethical concerns.

## Discussion

Our results confirm previous research which stated that parenthood is a very gendered experience ( Bower-Brown, 2022; Pakaluk & Price, 2020; Yaffe, 2023). Parenthood has a different meaning depending on the perceived gender of the parent. Women are perceived as more responsible for reproductive issues in general, and especially for the care for (young) children and household chores. They will probably remain at home to care for the children longer than men. Fatherhood is mostly associated with providing financially for the family. Research on the COVID-19 period indicates an intensification of this gender division in parenting ( Power, 2020). The gender division is often linked with presumed gendered biological differences. The assumption that in lesbian couples, parenthood is ‘more natural’, while male gay parents ‘do not know how to do it’ reflects these strong gender norms. 

In Belgium, Italy, Spain and Switzerland, the idea that women are responsible for reproductive issues conflicts with the idea of ‘motherhood as a choice’ and the belief that there should be gender equality in career opportunities. This paradox is reflected in the split subjectivity young women express, following the combination of a perceived gender equality in their professional career with unchanged traditional gender task division in private relationships ( O’Brien Hallstein, 2019). Participants of all genders express dissatisfaction with this gender inequality and feel that policy change is needed (e.g. better and more childcare options, equal paternity leave for both parents), as well as a changed attitude in individual fathers, hinting towards a more ‘caring’ masculinity ( Hunter *et al.*, 2017). One participant hints at the individualisation of childcare within the nuclear family and regrets there is no longer a community taking up part of the responsibility for caring for children. In Albania, Kosovo, North Macedonia and Slovenia, parenthood is perceived more as a duty for both men and women. Although participants in these countries also express that parenthood should be a choice, the duty belief seems to be stronger than the choice belief. Future research is needed to investigate the interaction of both beliefs and the meaning young people adhere to them.

An important result of our research is what Manon Vialle *et al*. call an ‘ambivalent relationship towards the procreative norm’ (unpublished): especially female participants express a desire to emancipate themselves from the procreative norm and at the same time reproduce that norm. The procreative norm, coined by Bajos and Ferrand (2006), specifies the "right" conditions for having a child as such: "a parental couple, emotionally, psychologically and materially stable, the birth being part of a parental project, and occurring at the right time in the professional trajectories of both parents" (2006: 92). In our research, finding ‘the right time’ is an important theme. Financial, professional and emotional stability are considered essential to start a family. However, combining family and career is a very gendered concern. Young women fear the professional implications of a pregnancy. They are aware of the discrimination towards mothers, even in countries where such discrimination is illegal (e.g. Van Hove, 2017). Stories of women being asked about their wish to start a family in job interviews, being less hired because they are assumed to become pregnant or fired once they are, all demonstrate the existence of normative discrimination of women at the workplace ( Benard & Correll, 2010). The accounts of participants carefully planning parenthood demonstrates their understanding of the economic reality. Their careful planning might indeed safeguard their economic status, as research confirms that delaying parenthood has positive effects on employment and income levels of young women in particular and a positive effect on the socioeconomic standing of parents of all genders ( Nisén *et al.*, 2022).

Vialle *et al.* (unpublished) state that the conditions of the procreative norm become rigid in certain aspects for these young people, while they become more malleable in others. For example, participants express the wish to have less stigma on infertility or voluntary childlessness, and many acknowledge that some people do not want to become parents, and support people in what they believe is a positive choice to not have children. However, some of their conceptions reconfirm a traditional procreative norm. They hold negative conceptions about childless people which reconfirm a gender essentialist belief that all women should become mothers, expressed in statements such as ‘a woman without children is a non-woman’. Our findings confirm the research by Colledge & Runacres (2023) where female participants in the UK (23–38 yo) demonstrated external positive prejudices against voluntarily childless women, while holding internal prejudices and biases.

Infertility is stigmatizing for all people since it is considered to make you less of a man or woman, but there are clear gender differences. In women, the stigma of infertility seems to be more linked to missing out on the practice of being a mother (whether biological or not), although some female participants also refer to pregnancy as an experience they do not want to miss out on. According to participants, the possibility of conceiving children in the traditional way confirms a man’s masculinity. A man’s fertility needs to be perceived as being intact, a result corresponding with the idea that manhood is seen as a ‘precarious state requiring continual social proof and validation’ ( Vandello *et al.*, 2008). Marcia C. Inhorn and Daphna Birenbaum-Carmeli argued that male infertility may be particularly problematic especially in Middle Eastern pronatalist societies, where ‘both virility and fertility are typically tied to manhood’ ( Inhorn, 2004, p. 162) or in non-Euro-American settings ( Birenbaum-Carmeli & Inhorn, 2009). Present research observes a similar trend in all investigated European countries. Birenbaum-Carmeli and Inhorn link the deep stigma on male infertility to the tendency to blame women for the infertility of the couple, even when the reproductive problem lies within the male body (2009, p. 24). Our research observes a similar co-occurrence of the stigma on male infertility and the trend to blame female bodies, sometimes leading to divorce, even if male factors are the reason for the couple’s infertility. This is a trend observed in many parts of the world (e.g. Ergin *et al*., 2018; Hasanpoor-Azghadi *et al*., 2019; Taebi *et al*., 2021) and our research’s value is in confirming a similar trend in the investigated countries.

Although participants argued that for men, it is not necessary to have children to be respected, most male participants affirmed their wish to become parents, thereby confirming that, contrary to stereotypes, fatherhood matters to men ( Hadley, 2019). Moreover, in many ways, our research confirms the findings of previous research (e.g. Bell, 2015) on similarities in gendered experiences of infertility. While infertility has physical and emotional implications on men as well as on women, an expectation expressed by many participants, ‘men’s reconciliations with those parallels silences their experience and perpetuates [reproductive] difference’ ( Bell, 2015). 

Another example of the ambivalence of young people regarding the procreative norm ( Vialle *et al.*, unpublished), is expressed in the wish of many participants to open up MAR techniques to LGBTQ+ people, while the ideal of a heterosexual two-parent family persists. In all countries, the acceptance of LGBTQ+ parenthood is quite high, even in participants who feel uneasy about homosexuality. Participants believe there is a general increase of acceptance towards LGBTQ+ people, which is in line with the 2019 Eurobarometer on Discrimination ( Directorate-General for Justice and Consumers of the European Commission, 2019). Only a small subset of our participants expressed outright homophobia, but the concern about the welfare of children in LGBTQ+ parent families is widespread. People worry about children of same-sex parent families becoming homosexual. This ‘fear of the queer child’ ( Rosky, 2013) already signals prejudices, since it is formulated as a concern. Another concern, extended to children in single parent families, is that ‘children need a mother and a father’ to develop into healthy adults, a persisting heteronormative prejudice despite a wide body of research evidencing the opposite (e.g. Golombok, 2022; Van Gelderen *et al*., 2012).

The most widespread concern focuses on the possibility that children of LGBTQ+ parent families might be bullied because of their family form, a concern present even in countries where LGBTQ+ parenthood is enshrined in law. This ‘benevolent’ concern can be seen as a form of harassment in itself, ‘enacted as a right to be concerned’ ( Ahmed, 2023, p.144). It influences LGBTQ+ participants insofar that they often don’t feel safe to raise a family because of possible unfavourable reactions and practical discrimination (e.g. De Wert *et al*., 2014; Perrin *et al*., 2019). This perception functions like a ‘door’ preventing LGBTQ+ people from starting a family: ‘The doors are not perceived by those who are not stopped by them. If you are stopped by a door, it can seem like you have stopped yourself’ ( Ahmed, 2023, p.165). With anti-gender discourse on the rise in Europe ( Kuhar & Paternotte, 2017, p.2), present research can contribute to mapping changing perceptions on LGBTQ+ parent families.

However, participants also believe that people for whom parenthood is not a given, like LGBTQ+ parents or single parents by choice, will be better parents because they have given parenthood much thought. The assumption is that those who find themselves in an exceptional situation, such as LGBTQ+ persons who want to become parents, might rise to the occasion and ‘overcome’ their difficulties to become an even better parent ( Golombok, 2015). This brings to mind the superhero narratives found in discourse on disability (the ‘supercrip’, e.g. Stanley, 2020) or motherhood (the ‘supermom’ – e.g. Magnus, 2020; O’Brien Hallstein, 2015). Superhero narratives emphasize the exceptionality of the situation and thus ‘other’ people, based on the assumption that a ‘normal’ person in this situation would not be able to do these things ( Schalk, 2016, p.79). Hence, rather than equalizing LGBTQ+ and straight parenthood, these narratives reaffirm the norm of the heterosexual parent couple.

While this research did not include many trans voices, they clearly signal that they feel left out regarding fertility. This confirms previous research signalling a lack of appropriate fertility counselling ( Rodriguez-Wallberg *et al*., 2023), leading to the assumption that fertility is only for cisgender people ( Wiesenthal *et al*., 2022), and the lack of cultural scripts regarding transgender parenting ( von Doussa *et al*., 2015). Moreover, next to restrictive adoption legislation in European countries ( ILGA Europe, 2023), research demonstrates that within Europe there is a variety of regulations regarding fertility treatment and associated issues like the documentation of parenthood in birth certificates and passports, making the journey to parenthood through MAR for trans, genderqueer and non-binary people ‘a fraught business’ ( Leibetseder & Griffin, 2020). The testimonies of trans people in this research prompt for more in-depth and intersectional research into trans and non-binary people’s experiences with MAR and parenting in Europe (see e.g. Goldberg, 2023, on the USA; Bower-Brown, 2022, on the UK; and Tam, 2021, on Canada).

## Limitations and methodological considerations

As rich and relevant as these findings are, it shows that scientific research, including this one, is still struggling to adequately integrate the diversity of genders and sexualities, to consider sexual minorities and to go beyond the heteronormative and binary representation of reproduction and parenthood. This is especially important in research considering encounters with the medical system, which often reproduces existing inequalities ( Goldberg, 2023; Tam, 2021). Despite ambitious research objectives, cis and heterosexual people were more present in the interview cohort than intended.

Moreover, we realise that binarity was still engrained in this project on several levels. First, in the gender categories, with every ‘trans’ category, ‘male to female’ or ‘female to male’ was added. Participants identifying as (non-binary) trans, genderqueer, non-binary, intersex or otherwise could be registered as ‘other’. This requires active participation of participants at the beginning of an interview, a moment when trust might not have already been established, especially in countries with low LGBTQ+ acceptance. Moreover, instead of the categories ‘female’ and ‘male’, ‘cisman’ and ‘ciswoman’ would have better fitted an inclusive approach. A similar limitation applies to the sexuality categories, where the options were heterosexual, homosexual, bisexual or other.

Second, the interviewers did not receive definitions of the categories, although there are many definitions of e.g. ‘transgender’, and the difference between transgender and transsexual is not clear. It is possible that participants identified differently and interviewers adjusted their answers to one of the categories they thought fit. In some cases, information gathered by the interviewer did not accord with the information disclosed during the interview, e.g. someone listed as bisexual talking about relations with trans and cis people (more commonly called ‘pansexual’); or someone listed as a heterosexual man told the interviewer he had relations with men ^9^. As analysing researchers, we had no contact with the interviewers, which led to interpretation difficulties and unclarity about the way information was obtained.

Third, the absence of certain variables, like intersex or non-binary, is remarkable. Multiple parenthood, e.g. in the case of a lesbian and gay couple raising a child together, or a lesbian couple with a known donor who wants to be involved in the life of the child, was only being discussed when participants talked about it themselves. In this way, traditional families take a central place in this research project. Moreover, because of the interplay of different axes of privilege and oppression in LGBTQ+ parents’ lives, a comprehensive gender approach needs to take other variables into account as well, like class, race, and disability (e.g. Goldberg, 2023). Because of this partial gendered approach to the initial research project and the resulting absence of certain variables, our gender analysis has some limitations. Still, we believe B2-InF brings interesting results, and we hope that this project will pave the way for further research on parenthood, infertility and MAR in Europe. The above mentioned methodological considerations may help future researchers develop a more in-depth and comprehensive gender approach.

## Ethical approval

This project (n°2021.004) received ethical approval on 29/01/2021 by the Research Ethics Committee of the University of Navarra. All interview participants received and signed informed consent forms. In case of in-person interviews, the consent form was signed physically; in case of online interviews, the consent form was sent in PDF format and returned signed by participants. The form stated that the transcripts would be used to prepare scientific articles and reports related to B2-InF. In addition, during the introduction of the interview, participants audio-recorded their consent to participate.

## Figures and Tables

**Table:** 

Conceptualization, funding acquisition, project administration, supervision	FG
Investigation (data collection) Investigation (first analysis) Investigation (in-depth analysis)	JMC, MLT, VD, ADBS, MF JMC, MLT, VD, ADBS, MF JS, MV, VR, KH
Resources	JMC, VD, ADBS, MF
Validation	All authors
Writing – Original Draft Preparation	JS
Writing – Review & Editing	KH, VR, MV, FG, JMC

## Data Availability

The data underlying the results cannot be shared for confidentiality reasons. The data has been restricted due to the sensitive nature of the information provided. There is a concern that the fertility industry might exploit this information for commercial purposes. Therefore, in the Grant Agreement nr. 872706 — B2-InF, signed by the Research Executive Agency of European Commission and all B2InF project partners, was established that the dissemination level of the interviews, as well as the dissemination level of the reports and thematic analyses of the interviews, is "confidential", explicitly defined as "only for members of the consortium (including the Commission Services)". (see Annex 1, p. 6/27 of the Grant Agreement nr. 872706). To request access, please email the corresponding author. Access will be granted once is confirmed that the data will be used solely for scientific research purposes and that all researchers involved in the study have no conflicts of interest. For the evaluation of the request, the Principal Investigator (PI) should submit: A draft that includes, at a minimum, the hypothesis, the objectives of the research project, and the methodological framework. A CV of the last 15 years for each researcher who will participate in the research and/or will have access to the data. A personal statement from each researcher addressing the following points separately: In my last 15 years, neither my institution nor I, personally, have received salary, funding, grants, or personal fees from entities funded by the fertility industry (including clinics, laboratories or biopharma companies). In my last 15 years, neither my institution nor I, personally, have currently or previously collaborated with advocacy groups or civil associations related directly or indirectly to fertility issues. I have never held a position on boards of scientific, social or commercial entities funded by the fertility industry (including clinics, laboratories, or biopharma companies). Zenodo: Gender aspects in parenthood conceptions of young Europeans. A critical reflection on the European Project B2-InF All data and materials are available on DOI https://zenodo.org/communities/b2inf_h2020/ ( Carrasco *et al.*, 2023). This project contains the following extended data: National guidelines for each country Global recommendation guidelines Policy briefs for each country Global policy brief Project communication (e.g. newsletters, flyers, conference posters…) Research project documents (e.g. reports, checklists, handbooks for data collection…) Scientific publications A DOI for this article will be sought in the same Zenodo community. DOI for similar scientific publication of the same project: 10.5281/zenodo.5704474 Data are available under the terms of the Creative Commons Attribution 4.0 International license (CC-BY 4.0).

## Footnotes

^1^ Ratio online/face-to-face interviews: ALB 2/7, BE 14/0, IT 15/0, KOS 0/15, MAC 9/0, SLO 10/0, SPA 7/8, SWI 10/0

^2^ Collected sociodemographic characteristics: age, gender, sexuality, occupation, education, residence, relationship, marital status, religion.

^3^ Legal analysis (Juana Farfán Montero, Marta María Albert Marquéz and María Rosa Tapia Sánchez, Universidad Rey Carlos, Madrid); sociocultural analysis (VR, MV); gender analysis (JS, KH).

^4^ Some authors are parents, others are not; some are heterosexual, some are not. Some of these positions may have influenced the analysis. JS, who was most involved in the presented gender analysis, is a lesbian researcher and mother, whose PhD research is on normative motherhood, mothering, feminism and maternal sense of agency.

^5^ The interviews were conducted in 2021, before Russia’s invasion of Ukraine in February 2022.

^6^ In July 2023, an prosecutor in Padua (Italy) demanded that lesbian co-mothers' names were erased from the birth certificates of their children. In March 2024, this policy was reversed by the Paduan court. The legal status of co-mothers remains insecure in Italy. See e.g. Franchi & Selmi (2020).

^7^ As for the countries surveyed, LGBTQ+ adoption is not legal in Albania, Kosovo, Italy and North Macedonia.

^8^ Italian and Spanish participants mention the pull of traditional norms, but stress that they do not adhere to these norms themselves.

^9^ This does not necessarily indicate an error or misunderstanding, since men who have sexual relationships with men (MSM) do not always identify as homosexual or bisexual ( Parrish, 2019). However, it means that these nuances have not been considered in this research.
