# Supplementary material for: Gender aspects in parenthood conceptions of young Europeans. A critical reflection on the European Project B2-InF: A critical reflection on the European Project B2-inf
Source: Open Res Eur. 2025 Feb 11;4:203. Originally published 2024 Sep 16. [Version 2] doi: 10.12688/openreseurope.18210.2 (PMC12576314; doi:10.12688/openreseurope.18210.2)
Supplement: Peer review history

# Reviewer report: Reviewer response for version 2

**DOI:** 10.21956/openreseurope.21243.r61527

**Published:** 2025-10-30

**Authors:** Kate Luxion

**Affiliations:** 1 University College London, London, England, UK

This article has the potential to expand the literature to better understand the topic within additional geographic contexts. However, there are some remaining issues that have been highlighted below. These should be interpreted as the need for significant changes in order to meet the scientific standard necessary for indexing

 The "Introduction" is presently reads as an extension to the project methods, so there is some contextualising information but this is not presented in the form of "What was known" prior to the study that would usually be expected for an article's introduction. Some of what is under "Selected Countries" could be reframed to better fit what is expected within an introduction.

 "who did not already have children or had used MAR" is a bit confusing - is this meant to mean that they had not already used MAR? Presently reads as not having children, but having used MAR. There are several clarity issues like this throughout the article.

 With how the methods are presently written there are sections that would be better suited for the introduction (e.g., the expectations of who is using more MAR) that currently make a more convuluted methods sections that could be read as blurring into results (which I don't think is the intention). Additionally, Table 1 and 2 should be within the results to define the sample and not within the methods, which shoud be dedicated to what was done.

 While in several of the key findings mention that "they felt it is still too early" to become parents or "From a certain age" there are social expectations, there is no mention of the age of the participants in the demographic tables to help contextualise this. While the age range is stated at the start of the methods, it would be best to have them repeated in the table, so that it is harder to miss this important detail.

 There is not always a clear integration of the study findings within the discussion. For example, there is mention of the assumptions of lesbian couples versus gay couples, but there is not a clarification if this seems to be confirmed within the study findings. Additionally, it feels like this is not included because it is also technically outside of the scope of the study to make a statement like this as the participants are not yet parents - so it should instead be framed as being about expectations and assumptions from the participants.

 Overall more significant changes deal with the reorganisation of the information to make sure that the structure of the article places the information into the expected portions of the paper structure (examples provided above). This should also help with some of the clarity issues that were mentioned as well.

If any, are all the source data and materials underlying the results available?

Partly

Is the work original in terms of material and argument?

Partly

Is the work clearly and cogently presented?

Partly

Is the argument persuasive and supported by evidence?

Partly

Does the research article contribute to the cultural, historical, social understanding of the field?

Partly

Does it sufficiently engage with relevant methodologies and secondary literature on the topic?

Partly

Reviewer Expertise:

LGBTQ+ parenting and reproduction, as well as broader issues that fall under the umbrella of reproductive justice

I confirm that I have read this submission and believe that I have an appropriate level of expertise to confirm that it is of an acceptable scientific standard, however I have significant reservations, as outlined above.

# Reviewer report: Reviewer response for version 2

**DOI:** 10.21956/openreseurope.21243.r51088

**Published:** 2025-02-21

**Authors:** Tanja Vuckovic Juros

**Affiliations:** 1 University of Zagreb, Zagreb, Croatia

I would like to thank the authors for their revisions. This was already a very good article in the previous draft, and I think that the revisions helped with transparency in relation to some methodological and analytical decisions.

 I admit I am somewhat disappointed that the authors did not explore more fully the possibilities to also revise and deepen their analysis and theoretical engagement. I truly believe that would have made this article much stronger. Nevertheless, this is authors' prerogative, and I will not insist on it.

 Overall, this is a very good article, and the revisions made it  more transparent analytically and therefore a better qualitative research article. I have no further reservations.

If any, are all the source data and materials underlying the results available?

Partly

Is the work original in terms of material and argument?

Yes

Is the work clearly and cogently presented?

Yes

Is the argument persuasive and supported by evidence?

Yes

Does the research article contribute to the cultural, historical, social understanding of the field?

Yes

Does it sufficiently engage with relevant methodologies and secondary literature on the topic?

Partly

Reviewer Expertise:

Qualitative research; Sociology of sexualities

I confirm that I have read this submission and believe that I have an appropriate level of expertise to confirm that it is of an acceptable scientific standard.

# Reviewer report: Reviewer response for version 1

**DOI:** 10.21956/openreseurope.19680.r45153

**Published:** 2025-01-07

**Authors:** Diego Lasio, Francesco Serri

**Affiliations:** 1 Universita degli Studi di Cagliari, Cagliari, Sardinia, Italy; 2 Dipartimento di Pedagogia Psicologia Filosofia, Universita degli Studi di Cagliari, Cagliari, Sardinia, Italy

We read this article on Gender Aspects in Parenthood Conceptions of Young Europeans with interest and pleasure. Since infertility and medically assisted reproduction are central topics, the article shifts focus toward people's relationships with social and health services and highlights the need to build democratizing processes for equitable access to these services. For this reason, we appreciate the space the article dedicates to methodological considerations regarding the representation of diverse perspectives on the explored topics, which are capable of moving beyond the heteronormative paradigm.

 We believe the article offers a valuable opportunity to observe—and in some cases confirm—trends in the investigated countries that have already been described in the literature regarding other nations, which constitutes its primary contribution.

 To better contextualize the data, it might be helpful to provide a brief yet comprehensive description of the local culture in which the research was conducted. We think it is important to address, from a gender perspective, the influence of pronatalist culture in various countries, as well as the cultural context that shapes practices concerning the division of caregiving responsibilities and the prevailing cultural meaning of parenthood (providing references).

If any, are all the source data and materials underlying the results available?

Yes

Is the work original in terms of material and argument?

Yes

Is the work clearly and cogently presented?

Yes

Is the argument persuasive and supported by evidence?

Yes

Does the research article contribute to the cultural, historical, social understanding of the field?

Yes

Does it sufficiently engage with relevant methodologies and secondary literature on the topic?

Yes

Reviewer Expertise:

Gender studies; Feminist theory; Sexualities; Intersectionality; Qualitative research; Discourse analysis.

We confirm that we have read this submission and believe that we have an appropriate level of expertise to confirm that it is of an acceptable scientific standard, however we have significant reservations, as outlined above.

# Response

**Published:** 2025-02-06

**Authors:** Joke Struyf

**Affiliations:** Philosophy, University of Antwerp Faculty of Arts, Antwerp, Flanders, Belgium

Thank you for the helpful suggestions.

 In this second version, we have considerably expanded the contextualisation of the findings, by providing additional sociodemographic and socioeconomic information and referring to relevant studies on gender and parenthood attitudes in the selected countries.

 We hope you find the changes sufficient.

# Reviewer report: Reviewer response for version 1

**DOI:** 10.21956/openreseurope.19680.r44473

**Published:** 2024-10-09

**Authors:** Tanja Vuckovic Juros

**Affiliations:** 1 University of Zagreb, Zagreb, Croatia

The article 'Gender aspects in parenthood conceptions of young Europeans' focuses on gender analysis of the data collected in 97 interviews with young people aged 18-30 in eight European countries. Its analysis examines in particular parenthood desires and expectations, the perceptions of the balance of family and career and the division of care, and the views on infertility. The results suggest the differences in perceptions that are linked to the perceived gender roles and, further, some country differences and ambivalences notwithstanding, many of these issues are similarly gendered across all eight participating countries.

 The article is clearly written and it is based on interesting and very valuable comparative data whose sheer scope, I suspect, makes the qualitative analysis a very demanding task. It is, therefore, completely understandable that any analysis must be in some way restricted. I think that the authors did a very good job of highlighting the main issues that they identified in their analysis. However, one element that I think could be strengthened more is a stronger contextualization of the research findings. This is the greatest strength of qualitative research compared to quantitative analyses, and it is an aspect that is all the more important for the data collected in eight different countries.

 Therefore, I would like to know more about the following issues:

- How were the eight countries selected? What is the most meaningful framework for comparing or clustering of these European countries specifically?
- Considering this is a gender analysis, how can these countries be contextualized in relation to gender attitudes and policies? This could be very, very brief, but I think it's something that should precede results.
- With these context differences in mind, how they might have affected some different patterns of findings in different countries, or different clusters of countries? Are there any patterns that stand out as similar across all countries, despite these countries' differences in gender attitudes and policies?
  - For example, there is a bit of this type of analysis in the section "The significance of the parenthood is gendered", but I would like to see this type of analysis done more systematically for all themes. It's also done slightly in the discussion, but again, I think that both the results and the discussion would benefit from more systematic contextualization.

The presentation of the methods and analysis is for the most part very clear, and I particularly appreciate the consideration of the diversity of participants and the section 'Limitations and methodological considerations' which I applaud for both its transparency and reflexivity. Still, some elements require greater attention in the text, to improve the transparency of methods and analysis even more. In particular:

- In line with the principles of Braun & Clarke's reflexive thematic analysis, I would like the authors to take more responsibility for their analysis. In particular, themes did not 'emerge', but somebody identified them, considered them, developed them? For example, based on footnotes, gender analysis seems done mostly by  JS and assisted by KH? It's not quite clear who and how (inductively, deductively, abductively?) developed the original code tree and what exactly did the development of 'own gender-related coding' involve? Was this mostly inductive process, based on the collected data, or something else?

The analysis is clear, mostly easy to follow and persuasive. However, I do think that it could be presented in a more systematic way to be even stronger in its implications and contributions. Specifically:

- I would also like to see more consideration of the validity of conclusions. Were there any validity checks done? For example, by looking for negative cases in the data, or some other method?  
- I would also be interested to hear more about how the patterns of findings hold up in terms of importance across countries, however 'importance' is interpreted by analysts. For example,  the important patterns of meaning might not be frequent, but they might be important precisely because they are usually assumed and therefore rarely mentioned. But sometimes, important patterns might be such because of the frequency. I'm asking this because I didn't get any sense of the difference in importance between various themes and patterns presented in the analysis. They were all mostly presented as "many participants believe", "many participants agree", "participants mention", etc. This is not a  problem in itself, it's a perfectly legitimate way to present the data, but I kept wondering were all these beliefs, agreements and mentions of the same importance, were they equally agreed upon/believed/mentioned by many in each of the countries, by each (gender or some other) profile of participants?

Finally, a minor comment which I don't think is a big issue, but I would like to mention nonetheless. Specifically, I was wondering if it was really necessary to base so much of the theoretical engagement on the unpublished work by one of the authors? While I understand completely that this article is building on earlier work of authors of the article (and even that there might not be much work on this), I think it's a good practice to engage more widely with other scholars concerned with similar issues. For example, in terms especially of LGBTQI+ parenthood and repronormativity, the work of Ana Cristina Santos comes to mind.  Therefore, as the article lacks a theoretical framework in the first part of the article, and only engages with the literature in the Discussion, I would suggest that the authors try to see if they could engage more widely with the other relevant studies and research, on gender attitudes if not on procreation norm (maybe this could also be connected to the stronger contextualization?).

 In conclusion, my strongest suggestions to the authors are to develop a more systematic presentation of results and a stronger contextualization of the data, and to add some more methodological details. However, I would also like to emphasize that these suggestions are meant to make this article even stronger (and I do hope authors will try to follow at least some of them), but this is already a very good and interesting article.

If any, are all the source data and materials underlying the results available?

Partly

Is the work original in terms of material and argument?

Yes

Is the work clearly and cogently presented?

Yes

Is the argument persuasive and supported by evidence?

Yes

Does the research article contribute to the cultural, historical, social understanding of the field?

Yes

Does it sufficiently engage with relevant methodologies and secondary literature on the topic?

Partly

Reviewer Expertise:

Qualitative research; Sociology of sexualities

I confirm that I have read this submission and believe that I have an appropriate level of expertise to confirm that it is of an acceptable scientific standard, however I have significant reservations, as outlined above.

## References

[1] : Gender aspects in parenthood conceptions of young Europeans. A critical reflection on the European Project B2-InF. *Open Research Europe* .2024;4: 10.12688/openreseurope.18210.1 10.12688/openreseurope.18210.1 38389664

# Response

**Published:** 2025-02-06

**Authors:** Joke Struyf

**Affiliations:** Philosophy, University of Antwerp Faculty of Arts, Antwerp, Flanders, Belgium

Thank you for the helpful suggestions and comments. We will answer point by point.

**On the selection of countries**:

 On the one hand, we were interested in having a heterogeneous sample from a geographical and socio-cultural perspective. On the other hand, we needed to select countries where we had partners and contacts to carry out the research. The selected countries meet those requirements. We have added this information to the article.

**On the contextualisation**:

 We have considerably expanded the contextualisation of the findings by providing sociodemographic and socioeconomic information and referring to relevant studies regarding gender stereotypes in the selected countries.

**On the methods and analysis**:

 We have provided more detailed information on coding and validation processes and rephrased some sentences to take more responsibility for the analysis.

**On patterns and themes:**

 The themes identified through reflexive thematic analysis were the same in all countries. However, we tried to be as specific as possible about differences between countries, by naming the countries instead of clustering them in ‘Northern’, ‘Southern’... We did not change this in the second version, as it was a conscious decision by the authors to be this specific. By using words like ‘many’ or ‘some’ participants, we tried to express the different weight of certain patterns.

**On references**:

 Following your suggestion, we have referred to relevant additional research which also helps to better contextualise our findings.

 We hope you find the changes sufficient.
